# Supplementary material for: End Sequence Analysis Toolkit (ESAT) expands the extractable information from single-cell RNA-seq data
Source: Genome Res. 2016 Oct;26(10):1397–410. doi: 10.1101/gr.207902.116 (PMC5052061; doi:10.1101/gr.207902.116)
Supplement: Supplemental Material [file supp_gr.207902.116_Supp_Materials.tar.gz › ESAT-esat_v0.1/doc/html/functions_vars_s.html]

ESAT: Class Members - Variables


ESAT


- Main Page
- Related Pages
- Packages
- Classes
- Files

- Class List
- Class Index
- Class Hierarchy
- Class Members

- All
- Functions
- Variables
- Enumerator

- a
- b
- c
- d
- e
- f
- g
- h
- i
- k
- l
- m
- n
- o
- p
- q
- r
- s
- t
- u
- v
- w


All Classes Namespaces Files Functions Variables Enumerator Pages

### - s -

- sample
  : broad.pda.seq.protection.WindowScores
- sampleModel
  : umms.core.model.score.MultiScore
- sampleName
  : broad.pda.seq.clip.SampleData
  , broad.pda.seq.protection.SampleData
- SAVED\_MODEL\_STATS\_EXT
  : broad.pda.seq.alignment.StoredAlignmentStats
- SCORE
  : broad.pda.seq.rap.GenomeScoringProgram
  , broad.pda.seq.rap.rna.TranscriptomeScoringProgram
- SEQ\_ID\_REC\_LENGTH
  : broad.core.sequence.FastaQualityBinaryIO
- sequenceDirectory
  : broad.pda.feature.genome.DirectoryInstalledGenomeAssembly
- SHORT\_ENCODED\_a
  : broad.core.sequence.Sequence
- SHORT\_ENCODED\_A
  : broad.core.sequence.Sequence
- SHORT\_ENCODED\_C
  : broad.core.sequence.Sequence
- SHORT\_ENCODED\_c
  : broad.core.sequence.Sequence
- SHORT\_ENCODED\_G
  : broad.core.sequence.Sequence
- SHORT\_ENCODED\_g
  : broad.core.sequence.Sequence
- SHORT\_ENCODED\_GAP
  : broad.core.sequence.Sequence
- SHORT\_ENCODED\_N
  : broad.core.sequence.Sequence
- SHORT\_ENCODED\_T
  : broad.core.sequence.Sequence
- SHORT\_ENCODED\_t
  : broad.core.sequence.Sequence
- SHORT\_READ\_FLOW
  : broad.core.sequence.Sequence
- shortBEDFactory
  : broad.core.annotation.AnnotationFactoryFactory
- signalData
  : broad.pda.seq.protection.TwoSampleScanSkellamPeakCaller
- signalName
  : broad.pda.seq.protection.TwoSampleScanSkellamPeakCaller
- signalProcessor
  : broad.pda.seq.protection.TwoSampleScanSkellamPeakCaller
- signalSamples
  : broad.pda.seq.protection.MultiSampleScanPeakCaller
- SIMILARITY
  : jaligner.Markups
- SIMPLE\_ARRAY\_LEFT
  : broad.pda.seq.rap.CountProbes
- SIMPLE\_ARRAY\_RIGHT
  : broad.pda.seq.rap.CountProbes
- SIZE
  : jaligner.matrix.Matrix
- sizeFile
  : broad.pda.seq.protection.MultiSampleScanPeakCaller
- SIZES
  : broad.pda.seq.rap.GenomeCommandLineProgram
- SORT\_ORDER
  : broad.pda.samtools.FilterPairedReadsByMAQ
- source
  : broad.core.annotation.BasicGenomicAnnotationReader
- SPLICED
  : broad.pda.seq.graph.ChromosomeWithBubblesJGraphT.EdgeSourceType
- START\_CODON\_PATRN
  : umms.core.annotation.Gene
- statistic
  : broad.pda.enrichment.EnrichmentMetric
- statisticName
  : broad.pda.enrichment.EnrichmentMetric
- STATS
  : broad.pda.seq.rap.CountProbes
- stepSize
  : broad.pda.seq.protection.MultiSampleScanPeakCaller
  , broad.pda.seq.protection.SampleData
  , broad.pda.seq.protection.TwoSampleScanSkellamPeakCaller
  , broad.pda.seq.protection.WindowScores
- STOP
  : jaligner.Directions
- STOP\_CODONS
  : umms.core.annotation.Gene
- SYMMETRIC
  : broad.pda.seq.rap.PlotAggregateRegions


---

Generated on Fri Aug 15 2014 15:11:17 for ESAT by  

 1.8.7
